# Mortality risk and years of life lost for people with reduced renal function detected from regular health checkup: A matched cohort study

**DOI:** 10.1016/j.pmedr.2022.102107

**Published:** 2023-01-03

**Authors:** Ta-Chien Chan, Yung-Hsin Chuang, Tsuey-Hwa Hu, Hugo Y.-H. Lin, Jing-Shiang Hwang

**Affiliations:** a Research Center for Humanities and Social Sciences, Academia Sinica, Taipei, Taiwan; b Institute of Public Health, School of Medicine, National Yang Ming Chiao Tung University, Taipei, Taiwan; c Institute of Statistical Science, Academia Sinica, Taipei, Taiwan; d Division of Nephrology, Department of Internal Medicine, Kaohsiung Medical University Hospital, Kaohsiung Medical University, Kaohsiung, Taiwan; e Department of Internal Medicine, Kaohsiung Municipal Ta-Tung Hospital, Kaohsiung, Taiwan; f Department of Medicine, College of Medicine, Kaohsiung Medical University, Kaohsiung, Taiwan

**Keywords:** eGFR, Proteinuria, Life expectancy, Hazard of death, Health check-up, ESRD, End-stage Renal Disease, CKD, Chronic Kidney Disease, LE, Life Expectancy, eGFR, estimated Glomerular Filtration Rate, TCR, Taiwan Cancer Registry, COD, Cause of Death, PS, Propensity Score, SMD, Standardized Mean Difference, SLED, Standardized Life Expectancy Deviation, YLL, Years of Life Lost, HR, Hazard Ratio, HTN, Hypertension, DM, Diabetes Mellitus, CVD, Cardiovascular Disease, CI, Confidence Interval, AFP, Alpha-fetoprotein, RAS, Renin-angiotensin System

## Abstract

With the increasing threat of metabolic syndromes, a focus on maintaining kidney health from early- to mid-adulthood is necessary. This study elucidates mortality risk and years of life lost (YLLs) due to abnormal renal function. This was a retrospective, matched cohort study from health checkup data from 2000 to 2015. We identified 12,774 participants with abnormal renal function (eGFR < 60 mL/min/1.73 m^2^) and used propensity score matching to identify 25,548 participants with normal renal function (eGFR ≥ 60). YLLs were estimated using the life expectancy differences between the abnormal and matched normal cohorts. Cox models were used to estimate the adjusted mortality risk. The estimated life expectancy of participants with proteinuria and eGFR < 60 was 26.24 years, with a 95 % confidence interval of (23.96, 29.36), 17.62 (16.37, 18.78), and 11.70 (11.02, 12.46) for age groups of 30 – 54, 55 – 64, and 65 – 79 years, respectively. The estimated YLLs of participants with proteinuria and eGFR < 60, as compared with the matched normal cohort, were 17.86 (13.41, 20.36), 12.55 (11.41, 13.78), and 8.31 (7.47, 9.13) years for the three age groups, respectively. The Cox model estimates of mortality hazard ratios of participants having proteinuria and eGFR < 60 against matched referents were 5.29 (3.97, 7.05), 3.99 (3.34, 4.75), and 3.05 (2.62, 3.55) for the three age groups, respectively. Abnormal renal function shortens life expectancy, particularly in patients with proteinuria and in younger adults. Active health management of renal function can reduce the disease burden.

## 1 Introduction

Renal function is known to decline with aging and is associated with a higher risk of progression to end-stage renal disease (ESRD) (Chuang et al., 2021, Hwang et al., 2010, Webster et al., 2017, Xu et al., 2019). It is life-threatening and often has high national medical expenses (Chan et al., 2014). Preserving renal function can prevent comorbidities (Couser et al., 2011) and complications. In 2019, kidney disease became the 10th cause of death, accounting for 1.3 million deaths worldwide according to WHO Global Health Estimates (https://www.who.int/news-room/fact-sheets/detail/the-top-10-causes-of-death). With rapid progress to an aging society and the increasing global epidemic of metabolic syndromes (Saklayen, 2018), a dose–response relationship between the traits of metabolic syndrome and the incidence of chronic kidney disease (CKD) was observed (Alizadeh et al., 2018). Patients with CKD, whether alone or combined with other chronic diseases, would have increased mortality risk, medical costs, and shortened life expectancy (LE) (Wan et al., 2019).

Taiwan has a high prevalence of CKD (11.9 %) and has the highest prevalence and incidence of ESRD in the world (https://adr.usrds.org/2020.AccessedMarch23/end-stage-renal-disease/11-international-comparisons); however, there was low public awareness, in which only 3.5 % of CKD patients were aware of their condition (Wen et al., 2008). Therefore, it is necessary to raise public and clinical awareness of CKD and its health impacts, including mortality risks and shortened LE. A population-based study from Canada found that a lower renal function level, represented by estimated glomerular filtration rate (eGFR) or proteinuria, is related to a shorter LE (Turin et al., 2013, Turin et al., 2012). Another Taiwanese prospective cohort study from 1994 to 2008 found a reduction in LE for early diabetic CKD patients (Wen et al., 2017). Two retrospective cohort studies in Hong Kong showed similar results, that is, people with severe CKD had higher medical costs and shortened LE (Wan et al., 2020, Wan et al., 2019). These studies applied either the abridged life table method or a flexible parametric survival model to calculate LE. Patients with more comorbidities had a higher risk for disease burden.

However, when comparing LE between different groups, only age and sex were considered, without accounting for other important factors that could also affect the lifespan. Statistical tests were performed to verify the presence of LE differences between groups. This study attempted to solve the above problems by matching the important factors, including age, sex, socioeconomic status (represented by educational attainment), and medical history; matching was performed between renal function groups to create study cohorts with abnormal renal function (eGFR and proteinuria) as well as matched normal cohorts. We further proposed to estimate a more accurate cohort LE instead of period LE, which is often calculated based on mortality rates in a few years to compare LE. The estimated LE of cohorts with and without abnormal renal function was compared separately for the three age groups. Finally, to explore the relationship between the shortened LE and mortality risk, we used Cox proportional models to estimate hazard ratios for the abnormal renal function groups with adjustment for potential risk factors.

## 2 Material and methods

### 2.1 Data and study population

**Fig. 1:**
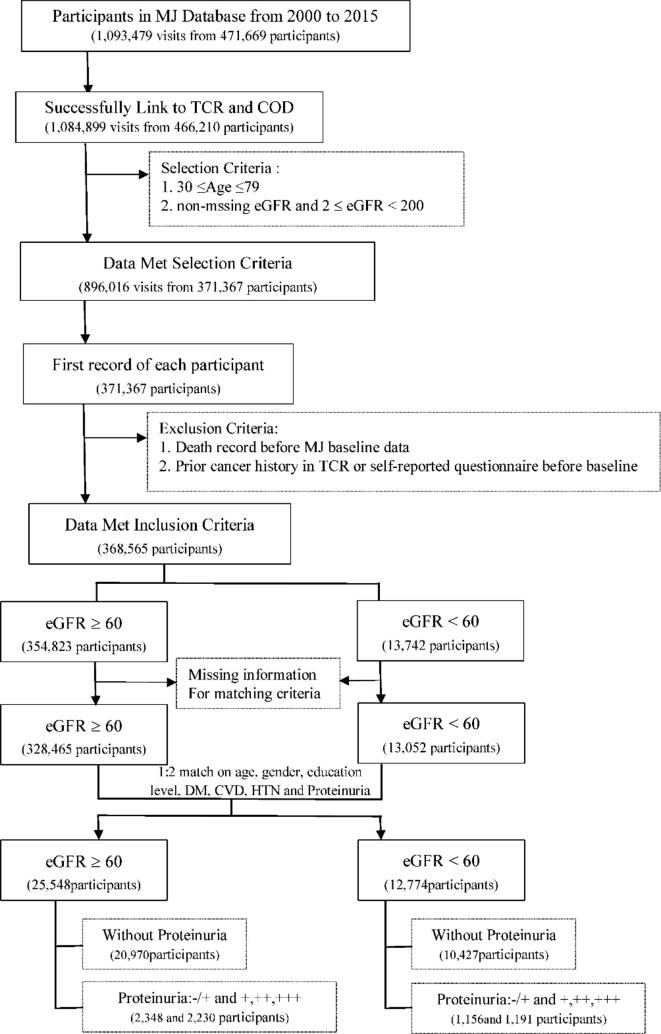
The flow chart of selecting participants.

During the 16-year study period, 471,669 people underwent health check-ups at the MJ Health Screening Center in Taiwan from 2000 to 2015. Information on lifestyle behavior was collected using a standard self-administered questionnaire. Anthropometric and biological data were collected during health examinations. This health database was linked to the cause-of-death dataset from 2000 to 2019 and the Taiwan Cancer Registry (TCR) from 2002 to 2015, with encrypted personal identification by trained staff members from the Health and Welfare Data Science Center, Ministry of Health and Welfare, and the MJ Health Research Foundation. The observed survival information (coded in ICD-9 and ICD-10) and cancer history (coded in ICD-O-3) were used to estimate LE and exclude participants with prior cancer history, either from TCR or self-reported cancer history, which could affect the estimation process. Fig. 1 shows the details upon data processing. As shown in Fig. 1, the health check-up data from 2000 to 2015 includes 471,669 participants and 1,093,479 records. Among them, 5,336 participants lacked identification information, and 123 had an incorrect information of birth year and gender compared with other participants registered in the health databases, such as the cause of death (COD) database and Taiwan cancer registry data (TCR). Thus, these patients were excluded, and 466,210 participants and 1,084,899 records that were linked successfully to TCR and COD were included. Further, we excluded 93,802 participants aged outside of our age range (30–79 years) and 2,825 participants with eGFR that did not range between 2 and 200, or with missing values. In total, 368,565 participants met the inclusion criteria. However, from the seven matching factors, five factors had missing values, including history of diabetes (0.77 %), cardiovascular disease (0.58 %), hypertension (0.58 %), proteinuria status (3.98 %), and education level (3.35 %). The 27,048 participants without matching factors were excluded before the matching process. Finally, 341,517 participants were included in the matching process, comprising 13,052 participants with eGFR < 60 and 328,465 participants with eGFR ≥ 60. For the three age groups (30–54, 55–64, and 65–79 years), a one-to-two matching process was applied based on the seven matching factors, for which 38,322 participants were finally included (eGFR < 60, 12,774 participants; eGFR ≥ 60, 25,548 participants) for further analysis.

The eGFR was calculated using the Chronic Kidney Disease Epidemiology Collaboration (CKD-*EPI*) equation (Levey et al., 2009). The date for the first eligible renal function measurement for each participant was set as the cohort entry date (baseline). Survival time was calculated from baseline, first checkup time, to the time of death, or censored at the end of 2019.

## 3 Study design and matching

We used a logistic regression model to conduct propensity score (PS) matching using the seven factors listed in Appendix 1. A matching process was conducted using greedy nearest-neighbor matching, which sequentially selected two persons from the normal group whose PS best matched the PS of one person from the abnormal groups. A caliper having a width of 0.2 was used to estimate the standard deviation of the logit of the PS, as suggested (Austin, 2011). The standardized mean difference (SMD) were used to examine the balance of matched factors between abnormal and normal groups. The threshold for declaring imbalance was SMD greater than 0.1 (Zhang et al., 2019). The above-mentioned matching processes were conducted using the “MatchIt” package in R 3.5.2. The participants in each of the three age groups were further stratified into six study cohorts using eGFR and proteinuria as the study indicators. Specifically, we compared five abnormal cohorts (eGFR ≥ 60, with trace proteinuria; eGFR ≥ 60, with positive proteinuria; eGFR < 60, without proteinuria; eGFR < 60, with trace proteinuria; and eGFR < 60, with positive proteinuria) with the normal cohort (eGFR ≥ 60, without proteinuria) to assess the mortality risk and burden of the disease. Due to the sample size limitation and the presence of data on healthy participants in the health check-up database, the number of participants with eGFR < 45, without proteinuria, and with trace proteinuria among participants aged 30–54 years were<100 participants, as was the number of participants aged 55–64 years with eGFR < 45 and with trace, and also those aged 65–79 years with eGFR ≥ 90 and with trace or proteinuria. Therefore, we conducted further analysis for the pooled age group of 30–79 years and stratified them into four eGFR groups including eGFR < 45, 45 ≤ eGFR < 60, 60 ≤ eGFR < 90, and eGFR ≥ 90.

The basic characteristics of the matched participants are presented using descriptive statistics. Differences in baseline characteristics between the two eGFR groups were assessed using independent t-tests for continuous variables and chi-square tests for categorical variables.

## 4 Estimation of LE

We found high percentages of censored survival times, even in the study cohorts with a maximum follow-up time of 20 years. One method to estimate the LE of a cohort was to extrapolate its survival function beyond the maximum follow-up time. We estimated the lifetime survival function of a cohort using a novel rolling extrapolation algorithm that has been successfully applied to several real-world problems of disease burden and cost-effectiveness assessment (Chang et al., 2021, Hwang and Hu, 2020, Hwang et al., 2017, Kuo et al., 2021; Wu et al., 2018a). The extrapolation method is briefly described in Appendix 2. The area under the estimated lifetime survival curve was the LE estimate of the cohort. The computations of LE, standard error, and 95 % confidence interval of the estimate were conducted using the R package iSQoL2 (R Core Team, 2019; Hu, 2019).

## 5 YLLs owing to abnormal renal function

To fulfill the primary aim, the MJ participants of each age group were classified into six study cohorts according to eGFR levels (eGFR ≥ 60 and < 60) and three types of proteinuria status (negative for no protein, trace [-/+] of protein found in urine, and positive for protein presented [≥+ sign] in urine). To compare LE among the study cohorts, we first adjusted for the possible effects of age and sex on LE by calculating the standardized LE deviation (SLED) of each cohort, which was the LE difference between the cohort and the age- and sex-matched reference generated from the same life tables in Taipei City. For each age group, the cohort with eGFR ≥ 60 and with no proteinuria was treated as normal. For the pooled age group, the MJ participants of each age group were classified into twelve study cohorts according to four eGFR levels and three types of proteinuria status. The cohort with eGFR ≥ 90 and with no proteinuria was treated as normal. We subtracted the SLEDs of the five abnormal cohorts from the normal to obtain the estimated expected years of life lost (YLL) for people with abnormal renal function.

## 6 Mortality risk of abnormal renal function

A Cox proportional model was used to explore possible mortality risk factors as well as to compare two renal function level and three proteinuria status groups among the three age groups and also for the pooled age group with the combination of four eGFR groups and three proteinuria status groups. The above procedures were conducted using the coxph function in R 3.5.2 (R Core Team, 2019); 19 risk factors were included in the Cox regression model (see Appendix 1). Finally, we compared the estimated YLL and hazard ratios (HRs) from the Cox proportional models of mortality risks of patients with abnormal renal function with adjustment for potential risk factors.

## 7 Results

There were 2,548, 4,196, and 6,030 participants with an eGFR < 60, as identified in the age groups of 30–54, 55–64, and 65–79 years, respectively. The numbers of matched participants with an eGFR ≥ 60 in the three age groups were 5,096, 8,392, and 12,060, respectively (Table 1). For the seven matching factors, including age, sex, education, hypertension (HTN), diabetes mellitus (DM), cardiovascular disease (CVD), and proteinuria, the SMD between the eGFR ≥ 60 and eGFR < 60 groups was < 0.1, indicating the balance of matched factors between the abnormal and normal groups (Table 1). The mean follow-up times were 179.07, 168.62, and 151.86 months for participants with eGFR < 60 in the three age groups (Table 2). For matched participants with eGFR ≥ 60, the mean follow-up times were 170.75, 164.31, and 154.91 months for the three age groups. Overall, the mortality rate was higher in the older age and poor renal function groups. Participants with an eGFR < 60 had a higher percentage of cerebrovascular and kidney diseases, gout, obesity, and higher blood lipid levels. In Table S1, we showed the descriptive statistics of eGFR by using data pertaining to the three age groups, abnormal or renal function, and three proteinuria statuses. The mean values of eGFR for abnormal function and proteinuria were 38.5, 37.8 and 40.0 for the age groups of 30–54, 55–64, and 65–79 years, respectively.

**Table 1:** The descriptive statistics of seven matching factors after propensity score matching between abnormal and normal renal cohort (SMD).

	**Age:**	**[30,54]**		**Age:**	**[55,64]**		**Age:**	**[65,79]**	
Variable	eGFR ≥ 60	eGFR < 60	SMD^a^	eGFR ≥ 60	eGFR < 60	SMD^a^	eGFR ≥ 60	eGFR < 60	SMD^a^
Total N	5,096	2,548		8,392	4,196		12,060	6,030	
Age: Mean(SD)	46.98(6.33)	46.85(6.38)	0.021	60.11(2.75)	60.11(2.74)	0.002	70.61(4.00)	70.71(4.07)	0.025
Male: N(%)	3,397(66.7)	1,681(66.0)	0.015	5,118(61.0)	2,526(60.2)	0.016	6,322(52.4)	3,225(53.5)	0.021
Educational Attainment: N(%)			0.017			0.012			0.030
Illiterate	91(1.8)	41(1.6)		680(8.1)	353(8.4)		2,387(19.8)	1,170(19.4)	
Under high school	2,578(50.6)	1,303(51.1)		6,070(72.3)	3,029(72.2)		8,067(66.9)	3,995(66.3)	
College or above	2,427(47.6)	1,204(47.3)		1,642(19.6)	814(19.4)		1,606(13.3)	865(14.3)	
Hypertension: N(%)	1,416(27.8)	715(28.1)	0.006	3,244(38.7)	1,619(38.6)	0.001	5,792(48.0)	2,929(48.6)	0.011
Diabetes mellitus: N(%)	538(10.6)	254(10.0)	0.019	1,668(19.9)	810(19.3)	0.014	2,509(20.8)	1,289(21.4)	0.014
Cardiovascular disease: N(%)	285(5.6)	162(6.4)	0.032	1,036(12.3)	541(12.9)	0.017	2,685(22.3)	1,377(22.8)	0.014
Proteinuria: N(%)			0.015			0.005			0.042
–	3,940(77.3)	1,962(77.0)		6,752(80.5)	3,368(80.3)		10,278(85.2)	5,097(84.5)	
-/+	432(8.5)	211(8.3)		763(9.1)	384(9.2)		1,153(9.6)	561(9.4)	
+/++/+++	724(14.2)	375(14.7)		877(10.5)	444(10.6)		629(5.2)	372(6.2)	

a Standardized mean difference (SMD).

**Table 2:** Characteristics of the included participants stratified by eGFR status at the baseline.

	**Age:**	**[30,54]**		**Age:**	**[55,64]**		**Age:**	**[65,79]**	
Variable	eGFR ≥ 60	eGFR < 60	P value	eGFR ≥ 60	eGFR < 60	P value	eGFR ≥ 60	eGFR < 60	P value
Follow-up time: Mean(SD)	170.75(52.95)	179.07(54.61)	<0.001	164.31 (55.99)	168.62 (57.07)	<0.001	154.91 (56.90)	151.86 (58.95)	0.001
Death: N(%)	280(5.5)	252(9.9)	<0.001	1,303(15.5)	562(21.2)	<0.001	4,492(37.2)	2,685(44.5)	<0.001
Smoking Status: N(%)			0.088			0.232			0.487
Never	3,266(64.1)	1,698(66.6)		5,786(68.9)	2,909(69.3)		8,754(72.6)	4,328(71.8)	
Former	440(8.6)	204(8.0)		842(10.0)	450(10.7)		1,450(12.0)	755(12.5)	
Current	1,390(27.3)	648(25.4)		1,764(21.0)	837(19.9)		1,856(15.4)	947(15.7)	
Drinking Habits: N(%)			<0.001			<0.001			<0.001
Never	3,799(74.5)	1,946(76.4)		6,162(73.4)	3,210(76.5)		9,560(79.3)	4,820(79.9)	
Former	190(3.7)	142(5.6)		508(6.1)	284(6.8)		812(6.7)	463(7.7)	
Current	1,107(21.7)	460(18.1)		1,722(20.5)	702(16.7)		1,688(14.0)	747(12.4)	
Obesity: N(%)	1,356(26.6)	659(25.9)	0.485	2,628(31.3)	1,394(33.2)	0.031	4,499(37.3)	2,374(39.4)	0.007
TG: N(%)	1,918(37.6)	1,087(42.7)	<0.001	3,104(37.0)	1,849(44.1)	<0.001	3,930(32.6)	2,463(40.8)	<0.001
HDLC_L: N(%)	1,118(21.9)	696(27.3)	<0.001	1,884(22.4)	1,188(28.3)	<0.001	2,946(24.4)	1,834(30.4)	<0.001
High AFP: N(%)	14(0.3)	5(0.2)	0.516	51(0.6)	9(0.2)	0.003	55(0.5)	22(0.4)	0.374
Long-term medication: N(%)	3,038(59.6)	1,249(49.0)	<0.001	3,544(42.2)	1,535(36.6)	<0.001	3,862(32.0)	1,654(27.4)	<0.001
Medicines for gout/ Uricosuric medicines: N(%)	155(3.0)	241(9.5)	<0.001	262(3.1)	374(8.9)	<0.001	380(3.2)	545(9.0)	<0.001
Medicine for high blood lipids: N(%)	192(3.8)	126(4.9)	0.015	388(4.6)	286(6.8)	<0.001	704(5.8)	404(6.7)	0.023
Cerebrovascular (stroke included): N(%)	41(0.8)	34(1.3)	0.027	159(1.9)	126(3.0)	<0.001	413(3.4)	234(3.9)	0.119
Kidney disease/Nephritis: N(%)	103(2.0)	254(10.0)	<0.001	141(1.7)	270(6.4)	<0.001	167(1.4)	278(4.6)	<0.001
Gout/Rheumatism: N(%)	399(7.8)	487(19.1)	<0.001	642(7.7)	699(16.7)	<0.001	908(7.5)	839(13.9)	<0.001

The estimated lifetime survival curves of the cohorts and those of the age- and sex-matched reference populations generated from the life tables of Taipei City for the three age groups are shown in Figure S1. Table 3 shows the estimated LE, which is the area under the lifetime survival curve of the study cohort according to age at enrollment. In the age group of 30–54 years, the LE of the cohort with eGFR < 60 without proteinuria was 39.65, with a 95 % confidence interval (CI) of (38.04, 40.77) years, which was lower than 41.63 (39.54, 42.68) years of the normal cohort. When the participants had proteinuria, LEs declined to 26.24 (23.96, 29.36) and 34.26 (31.12, 37.22) years depending on eGFR < 60 and eGFR ≥ 60, respectively, for this age group. For participants aged 55–64 years without proteinuria, LE of those with eGFR < 60 was 27.51 years, as compared to 29.51 years of those with eGFR ≥ 60. If they had proteinuria, LE decreased sharply to 17.62 years and 21.03 years for eGFR < 60 and eGFR ≥ 60, respectively. For participants without proteinuria and aged 65–79 years, LEs of those with eGFR < 60 and eGFR ≥ 60 were 18.21 years and 19.42 years, respectively. If they had proteinuria, LE decreased sharply to 11.70 years and 14.79 years for eGFR < 60 and eGFR ≥ 60, respectively. Regardless of eGFR and age group, the LEs of the population with trace proteinuria were intermediate between those with and without proteinuria.

**Table 3:** Estimated life expectancy from the enrollment stratified by age, proteinuria and eGFR status in the studied population.

		eGFR	≥60	eGFR	<60
Age	Proteinuria	LE	(95 % C.I.)	LE	(95 % C.I.)
[30,54]	–	41.63	(39.54, 42.68)	39.65	(38.04, 40.77)
	-/+	37.88	(34.46, 40.35)	31.30	(24.42, 36.66)
	+,++,+++	34.26	(31.12, 37.22)	26.24	(23.96, 29.36)
[55,64]	–	29.51	(28.98, 29.95)	27.51	(26.88, 28.18)
	-/+	24.60	(23.46, 25.75)	22.78	(21.34, 24.26)
	+,++,+++	21.03	(20.11, 21.96)	17.62	(16.37, 18.78)
[65,79]	–	19.42	(19.22, 19.64)	18.21	(17.98, 18.50)
	-/+	16.45	(15.90, 16.94)	14.57	(13.91, 15.32)
	+,++,+++	14.79	(14.18, 15.58)	11.70	(11.02, 12.46)

Table S2 lists the SLED estimates and 95 % CIs of the study cohorts. Overall, participants with eGFR < 60 and proteinuria had a significantly lower SLED than the reference population. The estimates of SLED were −13.89 (-16.24, −10.86), −9.12 (-10.38, −7.94), and −6.13 (-7.01, −5.23) years for the cohorts in the age groups of 30–54, 55–64, and 65–79 years, respectively. For participants with normal renal function, the estimates of the SLEDs were all positive, indicating that LE was better than that of the matched reference population.

The estimated YLL of the abnormal cohort was calculated by subtracting their SLED from that of the matched normal cohort. The estimated YLL values for the three age groups are presented in Table 4. For the cohort of participants with eGFR < 60 and proteinuria, the estimated YLLs were 17.86 (13.41, 20.36), 12.55 (11.41, 13.78), and 8.31 (7.47, 9.13) years among the 30–54, 55–64, and 65–79 years, respectively. For the cohort of participants with eGFR ≥ 60 and proteinuria, the estimated YLLs were reduced to 9.08 (5.18, 12.12), 9.01 (8.04, 10.11) and 5.06 (4.30, 5.73) years among the 30–54, 55–64 and 65–79 age groups, respectively. For the remaining abnormal cohorts without proteinuria, the estimated YLLs were much smaller and significant for the 55–64 and 65–79 age groups, with an estimate of 1.82 (1.23, 2.62) and 1.07 (0.62, 1.34) years, respectively. For participants with trace proteinuria, the YLLs were intermediate between those with and without proteinuria. As shown in Table 4, the estimates of all-cause mortality HRs of the abnormal cohort with both eGFR < 60 and proteinuria were 5.29 (3.97, 7.05), 3.99 (3.34, 4.75), and 3.05 (2.62, 3.55) for the 30–54, 55–64, and 65–79 age groups, respectively. For the cohorts with proteinuria and eGFR ≥ 60, the hazard ratios were reduced to 2.02 (1.48, 2.76), 3.03 (2.49, 3.69), and 2.02 (1.68, 2.43) for the three age groups, respectively. The estimated HRs for the remaining abnormal cohort without proteinuria for the three age groups were 1.39 (1.10, 1.77), 1.31 (1.17, 1.45), and 1.16 (1.09, 1.23), respectively.

**Table 4:** Estimated years of life lost (YLL) and hazard ratio (HR) of abnormal renal function cohorts compared to matched normal renal function cohorts stratified by age, eGFR status and proteinuria in the studied population.

				YLL		HR
Age	eGFR	Proteinuria	Estimate	(95 % C.I.)	Estimate	(95 % C.I.)
[30,54]	≥ 60	–		Baseline		Baseline
		-/+	3.46	(0.13, 6.88)	1.27	(0.87, 1.86)
		+,++,+++	9.08	(5.18, 12.12)	2.02	(1.48, 2.76)
	< 60	–	2.04	(-0.09, 3.98)	1.39	(1.10, 1.77)
		-/+	10.18	(4.31, 16.47)	2.28	(1.55, 3.34)
		+,++,+++	17.86	(13.41, 20.36)	5.29	(3.97, 7.05)
[55,64]	≥ 60	–		Baseline		Baseline
		-/+	4.48	(3.12, 6.04)	1.75	(1.49, 2.06)
		+,++,+++	9.01	(8.04, 10.11)	3.03	(2.49, 3.69)
	< 60	–	1.82	(1.23, 2.62)	1.31	(1.17, 1.45)
		-/+	6.43	(4.87, 7.98)	2.12	(1.74, 2.59)
		+,++,+++	12.55	(11.41, 13.78)	3.99	(3.34, 4.75)
[65,79]	≥ 60	–		Baseline		Baseline
		-/+	2.97	(2.28, 3.69)	1.46	(1.26,1.68)
		+,++,+++	5.06	(4.30, 5.73)	2.02	(1.68,2.43)
	< 60	–	1.07	(0.62, 1.34)	1.16	(1.09,1.23)
		-/+	4.87	(4.04, 5.54)	1.87	(1.65,2.12)
		+,++,+++	8.31	(7.47, 9.13)	3.05	(2.62,3.55)

Table S3 lists the personal risk factors identified in the Cox hazard models for the three age groups. Consistent significant risk factors were age, current smoking, higher alpha-fetoprotein (AFP), diabetes, and cerebrovascular disease. It is clear that the estimated hazard ratios were higher for participants with proteinuria and were even higher for those with both proteinuria and eGFR < 60.

In Table S4, there were 2,078 participants aged 30 to 79 years with eGFR < 45. The participant with poor eGFR tended to be older, male, more obese, and with shorten follow up time, and having higher mortality and more chronic diseases. The current smoking behaviors and drinking behaviors were higher in those participants with eGFR>=90. In Table S5, for the cohort of participants aged 30 to 79 years with eGFR < 45 and without proteinuria, the estimated YLL and hazard ratio were 4.18 (2.94, 5.33) years and 1.47 (1.28, 1.69), but if with proteinuria, the YLL and hazard ratio increased to 14.36 (12.91, 15.72) years and 4.85 (4.15, 5.66).

## 8 Discussion

This study illustrates a significant loss of life for people with abnormal renal function across three age groups by measuring two commonly used renal function indicators: eGFR and proteinuria. We found a strong relationship between mortality risk and YLLs with abnormal eGFR and proteinuria (trace or positive) from young adults to the elderly. Our study sheds light on the importance of actively managing renal function through regular health check-ups, which can be beneficial for preventing early life loss and reducing the overall disease burden.

In comparison with previous studies, one study in Canada found that compared with eGFR ≥ 60, LE was shorter at lower eGFR levels across all age groups and for both sexes (Turin et al., 2012). Another study presented that compared with those without proteinuria, people with mild (-/+ or 1 + ) and heavy (more than 2 + ) proteinuria also have shorter LE across all age groups and both sexes; YLLs for men and women aged 65 years with mild proteinuria are 4.1 and 5.5 years, respectively (Turin et al., 2013). The decreasing patterns of YLL with declining renal function in those studies were consistent with our findings.

Several characteristics of eGFR < 60 with proteinuria among the 30–54 years age group were noteworthy when compared with the rest of the age groups. Up to 23 % of CKD patients (i.e., eGFR < 60) among the 30–54 years age group had trace or proteinuria in the matched cohort, which indicated that early detection of proteinuria should be a cornerstone of CKD management. A serum creatinine test alone for eGFR may be inadequate for comprehensive renal function evaluation. The measurement of urinary protein levels using a urine dipstick is a simple method to achieve and provide more information about kidney health. Furthermore, YLL was significantly higher in the proteinuria group in all age groups. Additionally, given the same eGFR, patients with proteinuria had worse outcomes and higher mortality rates than those without. This result was in accordance with that of a Chinese study (Wu et al., 2018b), they suggested that proteinuria reflects glomerular and tubular dysfunction, is a significant risk factor for LE reduction, and is independent of eGFR.

This study has several strengths. First, this was a large health checkup cohort, highlighting that preventive medicine and active health management can be beneficial in reducing the disease burden. Second, this study had a relatively longer follow-up duration than similar studies. Validation of the robustness of our survival extrapolation algorithm within the cohort is important. Third, a matching process conducted before LE estimation was crucial to ensure comparability between abnormal and normal cohorts. This study not only considered age and sex, but also accounted for other important factors that may also affect lifespan.

However, this study also has some limitations. First, renal function indicators and other explanatory variables were measured at baseline; certain levels of misclassification may have occurred. Second, the risk of cancer affects the estimation of survival; hence, these participants were excluded from the data analysis. Additionally, we used two approaches to enhance comparability among different cohorts to extrapolate their survival function, that is, propensity matching and choosing the relevant reference population in Taipei City because the studied cohort might have had a higher socioeconomic status than the general population (Wu et al., 2017). Third, patients with advanced-stage CKD are rare among young adults; the data did not contain information on dialysis. Therefore, we could not further differentiate between the risk and YLL in patients with advanced CKD. However, the purpose of this study was to prevent the deterioration of renal function in CKD patients. The cut-off points of eGFR ≥ 60 and proteinuria are simple standards for screening patients in the clinical setting and in routine health check-ups. Fourth, the information on specific types of medications, such as renin-angiotensin system (RAS) blockade was unavailable in our data. However, we matched patients by the history of DM, CVD, and HTN, and stratified our analysis into three age groups and different renal functions. This should reduce the effect of a specific medication in each group. Fifth, the current health check-up data did not include information on the participants’ previous history of acute kidney injury or family history of kidney disease. Therefore, we could not adjust for these effects in our estimation. Sixth, the most commonly used indicators for checking renal function during health check-up are eGFR and the results from urine dipstick in Taiwan. As Taiwan has the highest incidence and prevalence of end-stage renal disease (ESRD) worldwide, early screening of the advanced stages of chronic kidney disease (CKD) is needed. As the clinical management of early CKD by the national health insurance system in Taiwan has advanced, the progression of CKD to ESRD has slowed down (Chan et al., 2014). Although the urine dipstick shows a high false positive rate ranging between 50 % and 90 % in other countries (Samal and Linder, 2013, White et al., 2011), our results nevertheless showed that proteinuria was consistently associated with poor life expectancy among different age groups, and even in the group with a good eGFR. Therefore, urine dipstick screening still plays an important role in health management. However, confirmation by using quantitative protein analysis is recommended for further clinical management.

## 9 Conclusion

Abnormal renal function shortens the estimated LE and is even worse in patients with proteinuria. Younger adults with abnormal renal function might have a higher mortality risk and longer YLLs. Thus, active management of renal function and improved health behaviors may be beneficial for these patients.

Disclosure

*Ethics approval and consent to participate*

Informed consent was obtained to authorize data processing and analysis. Ethical reviews were approved by the Institutional Review Board (IRB) of Biomedical Science Research, Academia Sinica (AS-IRB-BM-17044). Individually identifying data were removed and remained anonymous during the entire study.

*Availability of data and materials*

The data that support the findings of this study are available from the MJ Health Research Foundation and Ministry of Health and Welfare, Taiwan, but restrictions apply to the availability of these data, which were under approval for the current study and so are not publicly available. The linked data set used in this study had to be analyzed in person in the Health and Welfare Data Science Center, Ministry of Health and Welfare, Taiwan.

Funding

Funding: This research was supported by a grant from the Ministry of Science and Technology, Taiwan (MOST- 108–2628-M−001−008−MY3).

## Declaration of Competing Interest

The authors declare that they have no known competing financial interests or personal relationships that could have appeared to influence the work reported in this paper.

## Data Availability

The authors do not have permission to share data.
