# Supplementary material for: Mortality risk and years of life lost for people with reduced renal function detected from regular health checkup: A matched cohort study
Source: Prev Med Rep. 2023 Jan 3;31:102107. doi: 10.1016/j.pmedr.2022.102107 (PMC9938332; doi:10.1016/j.pmedr.2022.102107)
Supplement: Supplementary data 1 [file mmc1.docx]

# Appendix

## Appendix 1: Grouping explanatory variables

All explanatory variables for the Cox regression model were defined using baseline health examination data. These included age, sex, educational attainment, smoking status, drinking status, higher alpha-fetoprotein (AFP), higher triglyceride (TG), lower high-density lipoprotein (HDL-C), eGFR, proteinuria, long-term medication, medicines for gout/uricosuric medicines, medicines for high blood lipids, history of CVD, diabetes mellitus (DM), hypertension (HTN), cerebrovascular disease (stroke included), kidney disease/nephritis, gout/rheumatism, and obesity. The detailed definitions of variables were listed below:

### Self-administered questionnaire

**Demographic information:**

- - **(Matching factor #1) Age at enrollment** We treated it as a continuous variable in the Cox regression and classified it into three age groups (i.e., [30, 54], [55, 64], [65-79] years) when we computed life expectancy.
  - **(Matching factor #2) Sex** They were classified into two groups: males and females.
  - **(Matching Factor #3) Educational Attainment** They were classified into three groups: illiterate, high school or below, and college or above.

**Lifestyle behaviors at the time of study:**

- - **Smoking habits.** Classified into three groups: never, former, and current.
  - **Drinking habits.** Classified into three groups: never, former, and current.

**Medical history at the time of study:**

- - **Cerebrovascular (including stroke)** Defined by a reported history of cerebrovascular (including stroke) diagnosis Participants were classified into two groups: no and yes.
  - **Kidney disease/Nephritis** Defined by a reported history of kidney disease. Participants were classified into two groups: no and yes.
  - **Gout / Rheumatism.** Defined by a reported history of gout or rheumatism. Participants were classified into two groups: no and yes.

**Medication history at the time of study:**

- - **Long-term medication.** Defined by a reported history of long-term medication use. Participants were classified into two groups: no and yes.
  - **Gout/uric acid medicines** Defined by a reported history of medicine for gout Participants were classified into two groups: no and yes.
  - **Medicine for high blood lipid levels.** Defined by a reported history of medicines for high blood lipid levels. Participants were classified into two groups: no and yes.

### Body measurement

- **Obesity.** Abdominal obesity was defined as a waist circumference ≥ 90 cm (35 in) for men and ≥ 80 cm (31 in) for women. Participants were classified into two groups: no and yes.

### Blood test

- **High triglyceride (TG) levels.** Defined by whether the participant had a higher triglyceride level (TG ≥ 150 mg/dL) or current use of antitriglyceride drugs.
- **Low-density lipoprotein cholesterol (HDL-C).** Defined by whether the participant had lower HDL-C levels (HDL-C < 40 mg/dL for men and < 50 mg/dL for women)
- **High alpha-fetoprotein (AFP).** Defined by whether the participant had a higher AFP value (unit: ng/mL), which is a tumor marker, than normal. A normal AFP level of < 20. Participants were classified into two groups: no and yes.

### Urine test

- **(Matching factor 4) Proteinuria** An indicator of renal function is defined by dipstick urinalysis results. They were classified into two groups: normal for (-) and trace for (-/+), positive for (+), (++), (+++).

### Self-administered questionnaire + Blood Test

**Medical history at the time of study:**

- - **(Matching factor #5) Diabetes mellitus (DM).** Defined by either a reported history of DM diagnosis, current use of antihyperglycemic drugs in the questionnaire, or a record of fasting plasma glucose (FPG) ≥ level of 126 mg/dL during the health examination. Participants were classified into two groups: no and yes.
  - **(Matching factor #6) Cardiovascular disease (CVD).** Defined by a history of CVD diagnosis or current use of cardiac drugs. Participants were classified into two groups: no and yes.
  - **(Matching Factor 7) Hypertension (HTN)** Defined by a reported history of hypertension diagnosis or current use of antihypertensive drugs. Participants were classified into two groups: no and yes.

## Appendix 2: Estimating life expectancy of a cohort

First, the survival times for 100,000 individuals of a reference cohort that matched the sex, entry age, and entry year of the index cohort were generated using abridged life table of a relevant general population. These health checkup participants mainly came from the three largest cities in Taiwan; therefore, we used life tables of Taipei City for generating reference populations. The estimated survival function of the age- and sex-matched reference and index cohorts using the Weibull model are denoted as $S(t|r)$ for , and $S(t|i)$ for , respectively, where $F$ is the maximum follow-up and $L$ is the maximum lifespan (unit: months) since the entry age of the cohort.


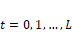

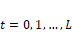

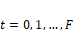

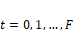


Second, we defined the relative survival function $W\left( t \right)=S(t|i)/S(t|h)$, where the survival curve $S\left( t | h \right)$ often equals $S(t|r)$ if $W\left( t \right)<1$. Otherwise, we used the survival curve with a proper value of the hazard parameter to ensure $W\left( t \right)<1$. The logit transformation of the relative survival function, denoted as $\mathrm{logit} W\left( t \right)$, is approximately linear after a certain follow-up period when the index cohort has an excessively constant mortality hazard. The approximate linearity makes the extrapolation of $\mathrm{logit} W\left( t \right)$ more robust for long-term predictions.


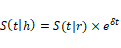

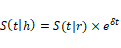

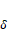

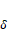


Third, a restricted cubic splines model was ﬁtted to the $\mathrm{logit} W\left( t \right)$ curve during the observation period to extrapolate the curve one time point ahead. The newly predicted value of $\mathrm{logit} W\left( t \right)$ at the neighboring time point, $\mathrm{logit} \hat{W}\left( F+1 \right)$, is usually highly accurate and treated as “new observation” at time $F+1$. The extrapolation procedures were then repeated by updating same-length observation periods one time point ahead and reﬁtting the restricted cubic spline models for the updated observation periods to predict the value of $\mathrm{logit} W\left( t \right)$ at successive time points until $t=L$.

Fourth, the lifetime survival function of the index cohort $\hat{S}\left( t | i \right)$could then be retrieved by

for .


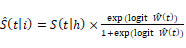

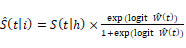

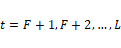

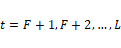


The life expectancy of the index cohort is the area under the extrapolated lifetime survival curve.

Figure S1-1. The survival curve of participants aged from 30 to 54 and the reference population in Taipei City from the enrollment


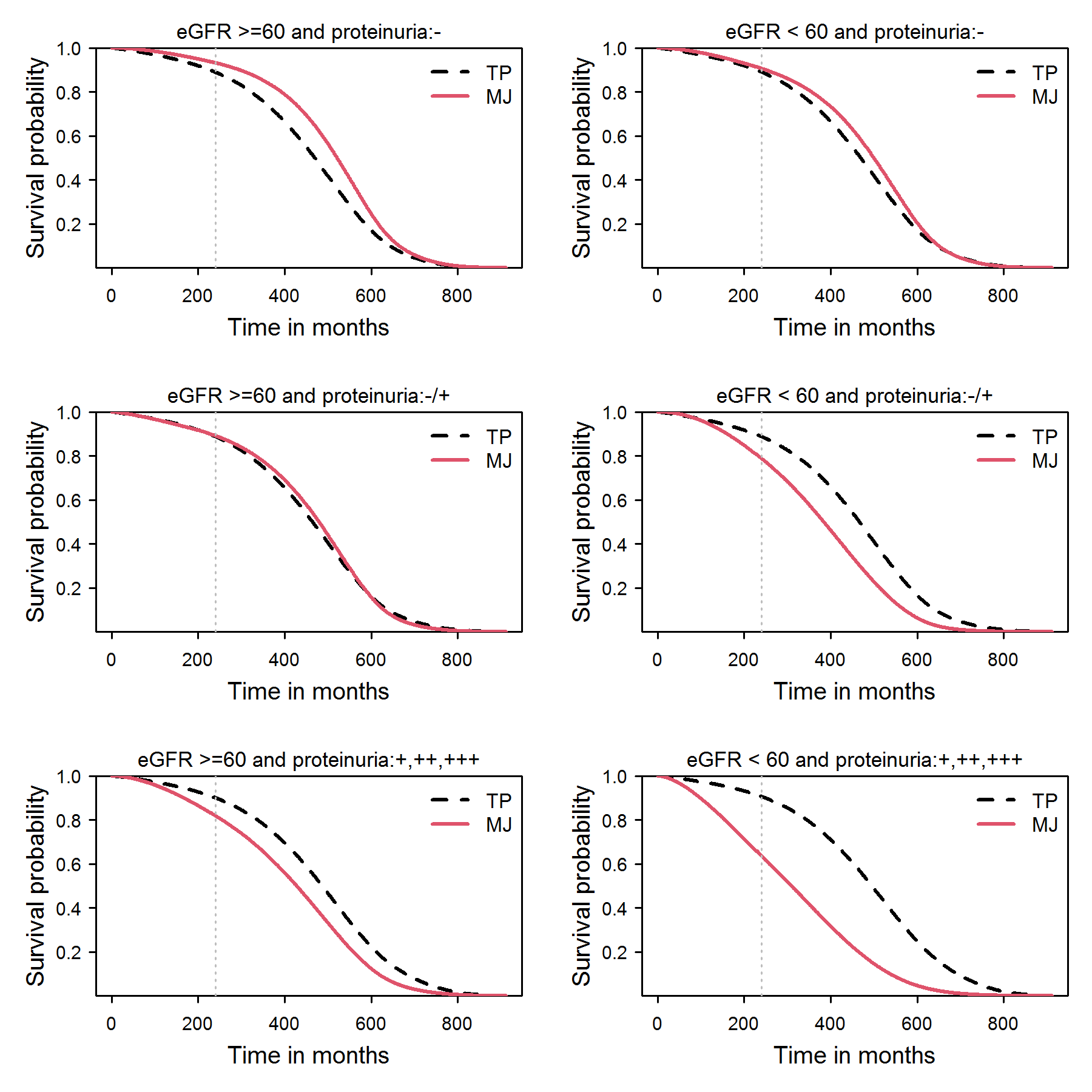


Legend of the figure:

TP stands for reference population in Taipei City.

MJ stands for the included participants from MJ health-up database.

Figure S1-2. The survival curve of participants aged from 55 to 64 and the reference population in Taipei City from the enrollment


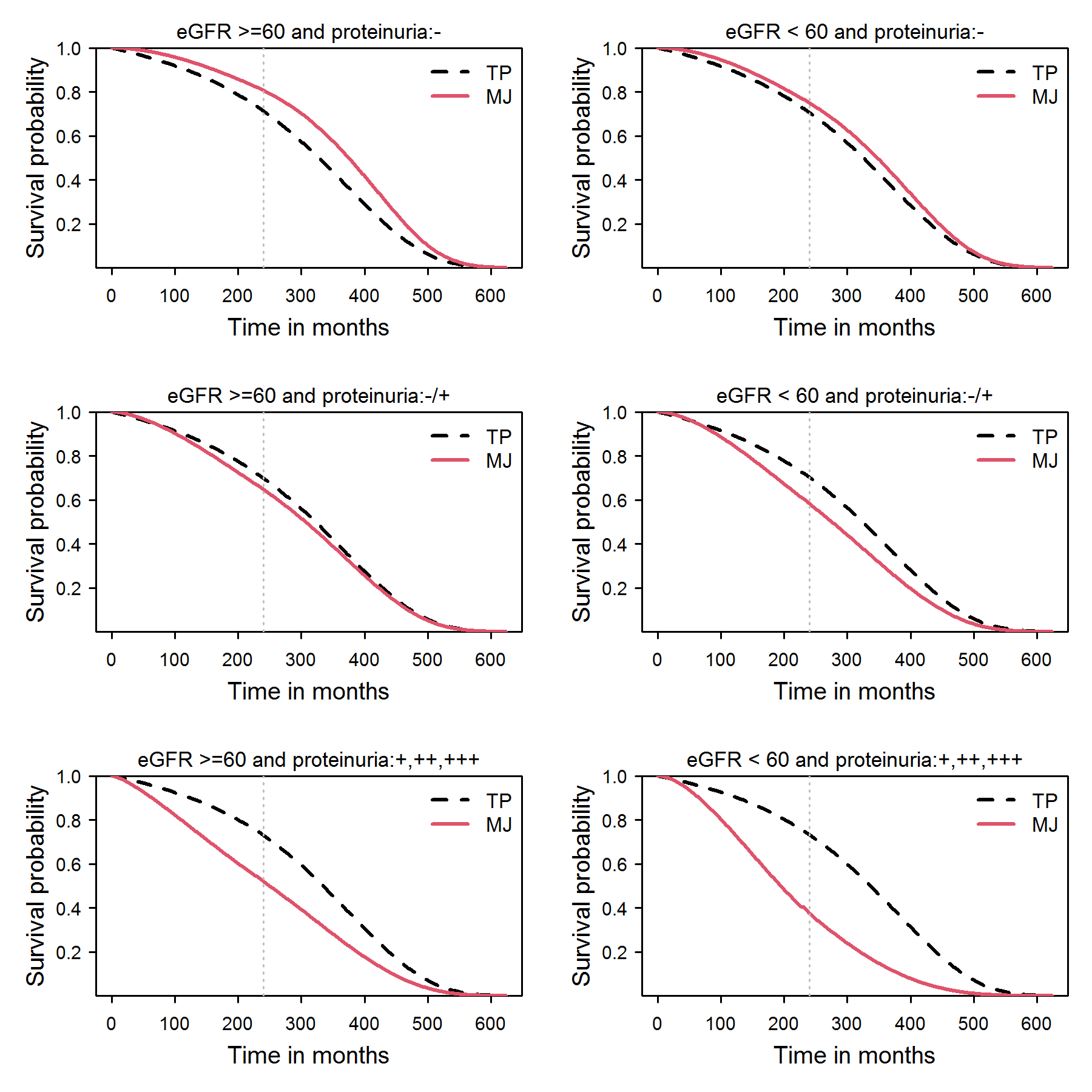


Legend of the figure:

TP stands for reference population in Taipei City.

MJ stands for the included participants from MJ health-up database.

Figure S1-3. The survival curve of participants aged 65 to 79, and the reference population in Taipei City from the enrollment


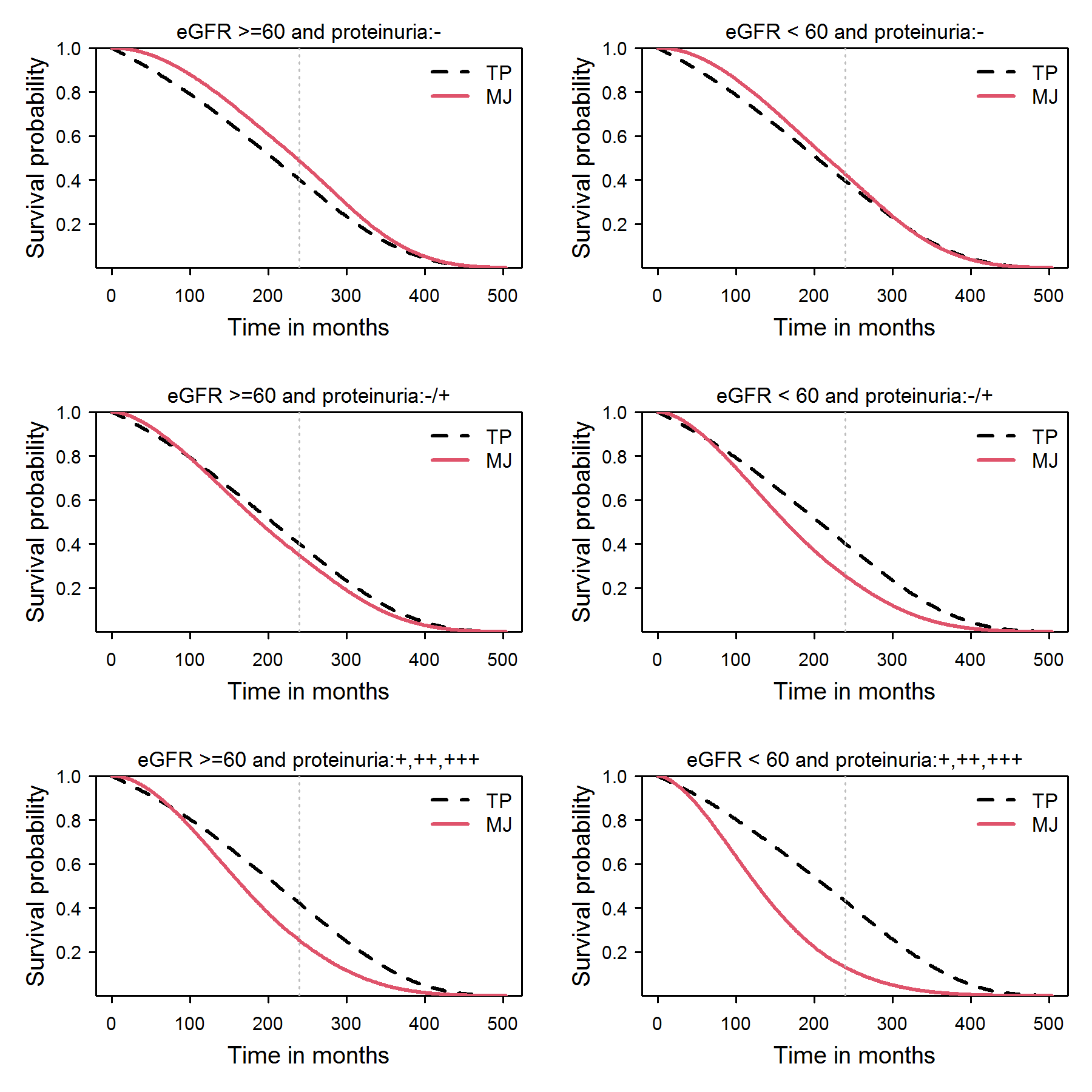


Legend of the figure:

TP stands for reference population in Taipei City.

MJ stands for the included participants from MJ health-up database.

Table S1. The statistical distribution of eGFR after propensity score matching stratified by age groups, eGFR groups and proteinuria groups

| Age | eGFR | Proteinuria | Mean | SD | Min. | 25% | 50% | 75% | Max. |
| --- | --- | --- | --- | --- | --- | --- | --- | --- | --- |
| 30-54 | ≥60 | - | 84.6 | 12.6 | 60 | 75.0 | 84.0 | 93.0 | 128 |
|  |  | -/+ | 83.9 | 13.2 | 60 | 74.0 | 84.0 | 92.0 | 122 |
|  |  | +/++/+++ | 83.5 | 14.7 | 60 | 71.0 | 83.0 | 95.0 | 123 |
|  | <60 | - | 54.9 | 5.8 | 4 | 54.0 | 57.0 | 58.0 | 59 |
|  |  | -/+ | 46.2 | 15.4 | 3 | 42.0 | 52.0 | 57.0 | 59 |
|  |  | +/++/+++ | 38.5 | 17.3 | 2 | 27.0 | 43.0 | 53.0 | 59 |
| 55-64 | ≥60 | - | 77.8 | 10.9 | 60 | 69.0 | 78.0 | 84.0 | 119 |
|  |  | -/+ | 76.5 | 11.1 | 60 | 67.5 | 73.0 | 83.0 | 112 |
|  |  | +/++/+++ | 76.0 | 12.0 | 60 | 66.0 | 73.0 | 83.0 | 124 |
|  | <60 | - | 53.1 | 6.1 | 5 | 51.0 | 55.0 | 58.0 | 59 |
|  |  | -/+ | 48.6 | 11.1 | 3 | 45.0 | 53.0 | 56.0 | 59 |
|  |  | +/++/+++ | 37.8 | 16.4 | 2 | 26.0 | 42.0 | 51.0 | 59 |
| 65-79 | ≥60 | - | 73.4 | 9.5 | 60 | 65.0 | 72.0 | 79.0 | 120 |
|  |  | -/+ | 72.3 | 9.3 | 60 | 65.0 | 71.0 | 77.0 | 106 |
|  |  | +/++/+++ | 73.3 | 9.7 | 60 | 65.0 | 71.0 | 78.0 | 99 |
|  | <60 | - | 51.6 | 7.1 | 12 | 48.0 | 54.0 | 57.0 | 59 |
|  |  | -/+ | 46.8 | 11.9 | 3 | 42.0 | 51.0 | 56.0 | 59 |
|  |  | +/++/+++ | 40.0 | 15.2 | 2 | 30.0 | 43.0 | 52.0 | 59 |

Table S2. Estimated standardized life expectancy deviation (SLED) between included participants and the reference population

|  |  | eGFR | ≥60 |  | eGFR | <60 |
| --- | --- | --- | --- | --- | --- | --- |
| Age | Proteinuria | SLED | 95% C.I. |  | SLED | 95% C.I. |
| [30,54] | - | 3.97 | (1.72, 4.95) |  | 1.94 | (0.25, 3.13) |
|  | -/+ | 0.52 | (-2.95, 2.88) |  | -6.21 | (-12.83, -1.00) |
|  | +,++,+++ | -5.10 | (-8.14, -2.15) |  | -13.89 | (-16.24, -10.86) |
| [55,64] | - | 3.43 | (2.93, 3.98) |  | 1.60 | (0.89, 2.24) |
|  | -/+ | -1.05 | (-2.40, 0.07) |  | -3.00 | (-4.56, -1.51) |
|  | +,++,+++ | -5.59 | (-6.63, -4.67) |  | -9.12 | (-10.38, -7.94) |
| [65,79] | - | 2.17 | (1.90, 2.35) |  | 1.10 | (0.87, 1.45) |
|  | -/+ | -0.80 | (-1.52, -0.30) |  | -2.70 | (-3.33, -1.86) |
|  | +,++,+++ | -2.89 | (-3.58, -2.15) |  | -6.13 | (-7.01, -5.23) |

*unit: years

^1^ SLED = LE of included participants – LE of reference population

Table S3. Personal risk factors of all-cause mortality hazard ratios

|  | Age: | [30, 54] | Age: | [55, 64] | Age: | [65, 79] |
| --- | --- | --- | --- | --- | --- | --- |
| Variable | HR | (95% C.I.) | HR | (95% C.I.) | HR | (95% C.I.) |
| **Age** | 1.06 | (1.03, 1.08) | 1.10 | (1.08, 1.12) | 1.11 | (1.10,1.12) |
| **Male** | 1.16 | (0.92, 1.47) | 1.43 | (1.26, 1.62) | 1.46 | (1.35,1.58) |
| **Educational Attainment:** |  |  |  |  |  |  |
| Under high school | 0.71 | (0.42, 1.19) | 0.74 | (0.64, 0.86) | 0.82 | (0.75,0.88) |
| College or above | 0.58 | (0.34, 1.01) | 0.55 | (0.45, 0.67) | 0.64 | (0.57,0.72) |
| **Smoking Status:** |  |  |  |  |  |  |
| Former | 0.91 | (0.65, 1.29) | 1.15 | (0.97, 1.36) | 1.16 | (1.06,1.28) |
| Current | 1.36 | (1.10, 1.69) | 1.66 | (1.47, 1.87) | 1.67 | (1.53,1.82) |
| **Drinking Habits:** |  |  |  |  |  |  |
| Former | 1.33 | (0.96, 1.85) | 1.27 | (1.06, 1.53) | 1.15 | (1.03,1.28) |
| Current | 1.30 | (1.04, 1.62) | 1.06 | (0.93, 1.20) | 0.90 | (0.83,0.98) |
| **Higher AFP** | 4.20 | (1.47,12.04) | 7.78 | (4.30,14.09) | 4.12 | (2.70,6.31) |
| **Long-term medication** | 0.65 | (0.52, 0.81) | 0.77 | (0.68, 0.85) | 0.86 | (0.81,0.93) |
| **Medicines for gout/Uricosuric medicines** | 0.97 | (0.66, 1.44) | 1.00 | (0.81, 1.24) | 1.04 | (0.92,1.18) |
| **Medicine for high blood lipids** | 0.82 | (0.57, 1.18) | 0.80 | (0.63, 1.01) | 0.77 | (0.67,0.89) |
| **Cardiovascular disease** | 1.29 | (0.97, 1.72) | 1.29 | (1.13, 1.46) | 1.16 | (1.08,1.24) |
| **Diabetes mellitus** | 1.92 | (1.52, 2.42) | 1.55 | (1.38, 1.74) | 1.38 | (1.28,1.48) |
| **Hypertension** | 1.24 | (1.00, 1.53) | 0.95 | (0.85, 1.06) | 0.99 | (0.93,1.06) |
| **Cerebrovascular (stroke included)** | 2.67 | (1.72, 4.14) | 2.22 | (1.84, 2.66) | 1.64 | (1.43,1.88) |
| **Kidney disease/Nephritis** | 0.98 | (0.68, 1.40) | 1.24 | (0.97, 1.58) | 1.17 | (1.00,1.37) |
| **Gout/Rheumatism** | 0.85 | (0.63, 1.15) | 0.91 | (0.76, 1.09) | 1.10 | (1.00,1.22) |
| **Obesity** | 1.05 | (0.86, 1.27) | 1.01 | (0.91, 1.12) | 1.04 | (0.98,1.11) |
| **TG** | 0.86 | (0.70, 1.06) | 0.95 | (0.86, 1.05) | 0.98 | (0.92,1.04) |
| **Lower HDL-C** | 1.18 | (0.96, 1.44) | 1.12 | (1.01, 1.24) | 1.03 | (0.96,1.10) |
| **(eGFR, Proteinuria):** |  |  |  |  |  |  |
| (<60, -) | 1.39 | (1.10, 1.77) | 1.31 | (1.17, 1.45) | 1.16 | (1.09,1.23) |
| (≥60, -/+) | 1.27 | (0.87, 1.86) | 1.75 | (1.49, 2.06) | 1.46 | (1.26,1.68) |
| (<60, -/+) | 2.28 | (1.55, 3.34) | 2.12 | (1.74, 2.59) | 1.87 | (1.65,2.12) |
| (≥60, +/++/+++) | 2.02 | (1.48, 2.76) | 3.03 | (2.49, 3.69) | 2.02 | (1.68,2.43) |
| (<60, +/++/+++) | 5.29 | (3.97, 7.05) | 3.99 | (3.34, 4.75) | 3.05 | (2.62,3.55) |

**AFP: alpha-fetoprotein; TG:** t**riglyceride; HDL-C: high-density lipoprotein cholesterol.**

Table S4. Characteristics of the included participants aged 30 to 79 years, stratified by eGFR status at baseline

| Variable | Value | eGFR:<45 | eGFR:45-59 | eGFR:60-89 | eGFR:≥90 | P value |
| --- | --- | --- | --- | --- | --- | --- |
| Total N |  | 2,078 | 10,696 | 21,504 | 4,044 |  |
| Age: Mean(SD) |  | 63.49(10.56) | 62.27(9.93) | 63.71(9.39) | 55.73(10.15) | <0.001 |
| Male: N(%) |  | 1,130(54.4) | 6,302(58.9) | 12,865(59.8) | 1,972(48.8) | <0.001 |
| Follow-up time: Mean(SD) |  | 144.51(61.95) | 166.35(57.16) | 159.9(56.76) | 167.84(52.54) | <0.001 |
| Death: N(%) |  | 1,030(49.6) | 2,795(26.1) | 5,433(25.3) | 642(15.9) | <0.001 |
| Smoking Status: N(%) |  |  |  |  |  | <0.001 |
| Smoking Status | Never | 1,494(71.9) | 7,441(69.6) | 1,4907(69.3) | 2,899(71.7) |  |
| Smoking Status | Former | 225(10.8) | 1,184(11.1) | 2,402(11.2) | 330(8.2) |  |
| Smoking Status | Current | 359(17.3) | 2,071(19.4) | 4195(19.5) | 815(20.2) |  |
| Drinking Habits: N(%) |  |  |  |  |  | <0.001 |
| Drinking Habits | Never | 1,648(79.3) | 8,328(77.9) | 16,382(76.2) | 3,139(77.6) |  |
| Drinking Habits | Former | 196(9.4) | 693(6.5) | 1341(6.2) | 169(4.2) |  |
| Drinking Habits | Current | 234(11.3) | 1,675(15.7) | 3,781(17.6) | 736(18.2) |  |
| Obesity: N(%) |  | 829(39.9) | 3,598(33.6) | 7,195(33.5) | 1,288(31.8) | <0.001 |
| TG: N(%) |  | 1,035(49.8) | 4,364(40.8) | 7,559(35.2) | 1,393(34.4) | <0.001 |
| Lower HDL-C: N(%) |  | 728(35.0) | 2,990(28.0) | 5,020(23.3) | 9,28(22.9) | <0.001 |
| High AFP: N(%) |  | 8(0.4) | 28(0.3) | 83(0.4) | 37(0.9) | <0.001 |
| Long-term medication: N(%) |  | 408(19.6) | 4,030(37.7) | 8,411(39.1) | 2,033(50.3) | <0.001 |
| Medicines for gout / Uricosuric medicines: N(%) |  | 337(16.2) | 823(7.7) | 719(3.3) | 78(1.9) | <0.001 |
| Medicine for high blood lipids: N(%) |  | 199(9.6) | 617(5.8) | 1,105(5.1) | 179(4.4) | <0.001 |
| Cerebrovascular (stroke included): N(%) |  | 119(5.7) | 275(2.6) | 565(2.6) | 48(1.2) | <0.001 |
| Kidney disease / Nephritis: N(%) |  | 417(20.1) | 385(3.6) | 361(1.7) | 50(1.2) | <0.001 |
| Gout / Rheumatism: N(%) |  | 473(22.8) | 1,552(14.5) | 1,757(8.2) | 192(4.7) | <0.001 |

Table S5. Estimated years of life lost (YLL) and hazard ratio (HR) of the abnormal renal function cohort compared to the matched normal renal function cohort stratified for eGFR status and proteinuria

|  |  |  | YLL |  | HR |
| --- | --- | --- | --- | --- | --- |
| Proteinuria | eGFR | Estimate | (95% C.I.) | Estimate | (95% C.I.) |
| - | ≥90 |  | Baseline |  | Baseline |
|  | 60-89 | -0.62 | (-1.63, 0.40) | 0.89 | (0.80, 0.98) |
|  | 45-59 | 0.15 | (-0.80, 1.25) | 1.01 | (0.91, 1.13) |
|  | <45 | 4.18 | (2.94, 5.33) | 1.47 | (1.28, 1.69) |
| -/+ | ≥90 | 4.87 | (1.92, 7.25) | 1.30 | (1.00, 1.70) |
|  | 60-89 | 3.25 | (2.16, 4.32) | 1.34 | (1.16, 1.55) |
|  | 45-59 | 4.54 | (3.24, 5.75) | 1.46 | (1.26, 1.71) |
|  | <45 | 7.93 | (6.03, 10.34) | 2.63 | (2.17, 3.19) |
| +,++,+++ | ≥90 | 9.17 | (6.95, 11.89) | 3.09 | (2.35, 4.04) |
|  | 60-89 | 7.21 | (6.00, 8.50) | 2.00 | (1.70, 2.35) |
|  | 45-59 | 9.17 | (7.29, 11.38) | 2.14 | (1.78, 2.57) |
|  | <45 | 14.36 | (12.91, 15.72) | 4.85 | (4.15, 5.66) |
